# Human Mesenchymal Stem Cells Differentiation Regulated by Hydroxyapatite Content within Chitosan-Based Scaffolds under Perfusion Conditions

**DOI:** 10.3390/polym9090387

**Published:** 2017-08-23

**Authors:** Anamarija Rogina, Maja Antunović, Lidija Pribolšan, Katarina Caput Mihalić, Andreja Vukasović, Alan Ivković, Inga Marijanović, Gloria Gallego Ferrer, Marica Ivanković, Hrvoje Ivanković

**Affiliations:** 1 Faculty of Chemical Engineering and Technology, University of Zagreb, Marulićev trg 19, p.p.177, 10001 Zagreb, Croatia; mivank@fkit.hr (M.I.); hivan@fkit.hr (H.I.); 2 Faculty of Science, University of Zagreb, Horvatovac102a, 10001 Zagreb, Croatia; maja.antunovic@biol.pmf.hr (M.A.); lidija.pribolsan@gmail.com (L.P.); katarina.caput.mihalic@biol.pmf.hr (K.C.M.); ingam@biol.pmf.hr (I.M.); 3 Department of Histology and Embryology, School of Medicine, University of Zagreb, Šalata 3, 10001 Zagreb, Croatia; andreja.vukasovic@mef.hr (A.V.); alan.ivkovic@gmail.com (A.I.); 4 Department of Orthopaedic Surgery, University Hospital, Sveti Duh, 10001 Zagreb, Croatia; 5 Centro de Biomateriales e Ingeniería Tisular, Universitat Politècnica de València, Camino de Vera s/n, 46022 Valencia, Spain; ggallego@ter.upv.es; 6 Biomedical Research Networking centre in Bioengineering, Biomaterials and Nanomedicine (CIBER-BBN), Mariano Esquillor s/n, 50018 Zaragoza, Spain

**Keywords:** chitosan, hydroxyapatite, hMSC’s, perfusion-bioreactor

## Abstract

The extensive need for hard tissue substituent greatly motivates development of suitable allogeneic grafts for therapeutic recreation. Different calcium phosphate phases have been accepted as scaffold’s components with positive influence on osteoinduction and differentiation of human mesenchymal stem cells, in terms of their higher fraction within the graft. Nevertheless, the creation of unlimited nutrients diffusion through newly formed grafts is of great importance. The media flow accomplished by perfusion forces can provide physicochemical, and also, biomechanical stimuli for three-dimensional bone-construct growth. In the present study, the influence of a different scaffold’s composition on the human mesenchymal stem cells (hMSCs) differentiation performed in a U-CUP bioreactor under perfusion conditioning was investigated. The histological and immunohistochemical analysis of cultured bony tissues, and the evaluation of osteogenic genes’ expression indicate that the lower fraction of in situ formed hydroxyapatite in the range of 10–30% within chitosan scaffold could be preferable for bone-construct development.

## 1. Introduction

The development of functional allogeneic tissue constructs requires the coordination of cell adhesion, growth, differentiation, and organization into specific tissue architecture. Accordingly, the engineering of a suitable stem cell microenvironment becomes an important approach in regenerative medicine [1]. Mesenchymal stem cells (MSCs) isolated from adult bone marrow are recognized as self-renewing cells capable of differentiating into multiple different cell phenotypes, providing many advantages in tissue regeneration strategies [2,3]. Even with precise control over materials characteristics at the nanoscale, there is still a challenge to control the protein adsorption and cellular response due to the complexity of biological systems [4].

Biomaterial scaffolds and dynamic bioreactors, such as perfusion bioreactor, represent two important components in the formation of suitable microenvironments providing controlled patterns of biochemical and biomechanical signaling [5]. Finding the proper synergy between scaffolds features and perfusion conditioning can direct MSCs behavior during the graft development.

Predominant factors in MSCs signaling are the scaffold’s composition, its surface properties, and specific cell-scaffold interaction that occurred during the implant-native tissue bonding [6]. Hydroxyapatite (HA) has already confirmed its osteoinductive properties in vitro and in vivo [7,8,9,10,11]. Chemical similarity to natural bone component makes it suitable for bone tissue integration and regeneration. Chitosan-based composites exhibit excellent properties: biodegradability, ability to support the bone tissue formation, possibility for modifications for specific cellular responses, inhibition of microorganisms, and oral pathogens [12,13,14,15]. Specific cationic nature of chitosan, depending on the surface modification, can positively influence protein and cell adsorption, allowing for good cell adhesion and migration, while its hydrogel properties ensure high water absorption which is important for fluid retention and nutrients transport in the biological environment. Osteogenic properties of chitosan/hydroxyapatite-based scaffolds have been previously reported by the long-term culture of human MSCs [16]. Promoted initial hMSCs adhesion on chitosan-gelatin/hydroxyapatite scaffold has maintained a higher progenicity and multi-lineage differentiation potentials with enhanced osteogenesis [6]. Osteogenic differentiation of hMSCs has been promoted by incorporation of hydroxyapatite nanoparticles into chitosan-silk fibroin matrix, as reported by Lai and Shalumon [17]. The influence of the hydroxyapatite amount was detected by extended differentiation for composites with a higher HA fraction.

The cell diffusion, cellular behavior, and tissue integration depend on scaffolds porosity, pore size, and overall pore interconnectivity. High porosity plays an important part in cell seeding, allowing for good cell distribution and tissue penetration [18]. Considerable influence of pore structure on effective diffusivity and bone ingrowth was reported by Jones et al. [19], indicating that the scaffold’s porosity should also be considered and optimized in the graft engineering.

Despite scaffolds’ requirements, instructive developmental stimuli can be provided by dynamic cell culture using perfusion bioreactors [20,21]. Study performed by Kim and Ma [20] has suggested that transversal media flow in the bioreactor culture of hMSCs accelerates progression from uncommitted mesenchymal stem cells, to osteoblasts as a combined result of shear stress stimulation and the depletion of mitogenic growth factors and ECM. The direct perfusion ensures exposure of the cells to interstitial fluid within the scaffold, resulting in higher seeding efficiency and even distribution of cells and newly formed tissue.

Apart from the extensive research of hydroxyapatite impact on MSCs differentiation, most of those investigations were performed in static cell cultures. According to our previous study [22], present materials have demonstrated very good osteogenic potential in the static cell culture of MC3T3-E1 preosteoblasts. In the current study, we wanted to confirm this behavior under dynamic hMSCs culture by eliminating possible hindrance in cell seeding of static conditions caused by differences in surface properties of composite scaffolds. We believe that biomechanical forces accomplished by media perfusion enhance seeding and distribution of the cells, as well as homogeneous distribution of macromolecules ensuring necessary microenvironment for hMSCs osteogenesis through a scaffolds’ interior. This preliminary study indicates that the lower fraction of hydroxyapatite within chitosan-based scaffold is favorable for allogeneic graft development.

## 2. Materials and Methods

### 2.1. Materials Preparation

Chitosan (*M*_w_ = 100–300 kg/mol, DD = 0.95–0.98, Acros Organics, Geel, Belgium), calcium carbonate (CaCO_3_, calcite; TTT, Sv. Nedjelja, Croatia), urea phosphate ((NH_2_)_2_CO–H_3_PO_4_; Aldrich Chemistry, St. Louis, MO, USA), acetic acid (HAc; POCH, Gliwice, Poland), sodium hydroxide (NaOH, Carlo Erba, Val de Reuil, France), and ethanol (EtOH, 96 wt %, Kefo, Sisak, Croatia) were all of analytical grade.

The synthesis of chitosan-hydroxyapatite (Cht-HA) composites with different amount of hydroxyapatite precursors, as well as porous structures, was done according to our previous work [23]: to obtain required HA percentage, appropriate amounts of calcite were suspended in chitosan solution (1.2 wt %) prepared in dilute acetic acid solution (0.36 wt %) at an ambient temperature. Following additions of a specific amount of urea phosphate, with respect to the Ca/P ratio of 1.67, characteristic for hydroxyapatite. Temperature was set at 50 °C and the precipitation reaction was continued for 4 days. The precursor’s amount was adjusted to obtain 0, 10, 30, and 50 wt % of in situ precipitated HA in final Cht-HA scaffold.

The production of porous chitosan-hydroxyapatite structures was initialized by cooling down the suspensions to room temperature, and freezing them over night at −22 °C. Frozen samples were immersed into different mediums, starting with a neutralization medium consisting of equal volume portions of 1 mol/L NaOH and EtOH, and then pure ethanol (96 wt %) at −22 °C. Finally, samples were dehydrated with ethanol at an ambient temperature and left out to dry for 24 h.

### 2.2. Scaffolds Characterization

#### Microstructure Imaging

The composite Cht-HA scaffolds were imaged by the scanning electron microscope TESCAN Vega3SEM Easyprobe at electron beam energy 10 keV. Previously to imaging, samples were sputter coated with gold and palladium for 120 s.

### 2.3. Dynamic hMSCs Cell Culture in Perfusion Bioreactor

#### 2.3.1. Human Mesenchymal Stem Cells Isolation and Expansion

The bone marrow sample was collected during the surgery at the University Hospital of Traumatology in Zagreb, Croatia, with the patient’s consent and approval of the Ethics Committee. The hMSCs were isolated using previously described method [24]. Briefly, 20 mL of bone marrow aspirates were added to 200 mL Dulbecco’s modified Eagle medium with 1000 mg/L glucose (DMEM-low glucose) (Lonza, Basel, Switzerland) containing 10% fetal bovine serum (FBS) (Gibco, Gaithersburg, MD, USA), 100 U/mL penicillin and 100 µg/mL streptomycin (Lonza). The suspension was centrifuged at 300× *g* for 10 min, and pelleted cells were washed twice in phosphate-buffered saline (PBS) (Gibco). Resuspended cells were strained through a cell strainer (100 μm) (BD Biosciences, Mississauga, ON, Canada) to remove bone chips and then centrifuged at 300× *g* for 10 min. Cells were plated in 100 mm Petri dishes (Sarstedt, Germany) at a density of 1 × 10^8^ in proliferation medium DMEM-low glucose, supplemented with 10% FBS, 100 U/mL penicillin and 100 µg/mL streptomycin and 10 ng/mL human fibroblast growth factor 2 (FGF2) (Gibco), and kept in a humidified incubator at 37 °C with a 5% CO_2_ supply. After 24 h the non-adherent cells were removed by total media replacement, and the attached cells were grown. When hMSCs became 80% confluent, they were detached with 0.25% trypsin/EDTA (Sigma-Aldrich) and then subcultured for expansion. Proliferation medium was changed every 2–3 days. After 24 h, the number of attached hMSCs has been determined by counting the number of cells in medium (unattached cells).

#### 2.3.2. Osteogenic Induction in Perfusion Bioreactor

Perfusion bioreactor (U-CUP Cellec Biotek, Basel, Switzerland) was used for three-dimensional bone tissue growth. Two samples of each scaffold, with diameter of 8 mm and height of 2 mm, were inserted into the bioreactor for cell seeding with a cell suspension of 1.6 × 10^6^ cells/bioreactor. The bioreactor chamber was closed and a bioreactor with a perfusion speed of 1.7 mL/min was placed in an incubator at 37 °C and 5% CO_2_ for 24 h.

The osteogenic induction medium (Minimum Essential Medium-Alpha Eagle (α-MEM) (Lonza), 10% FBS, 1% penicillin/streptomycin, 50 µg/mL ascorbic acid (Sigma-Aldrich), 4 mmol/L β-glycerophosphate (Sigma-Aldrich) and 1 × 10^−7^ mol/L dexamethasone (Sigma-Aldrich) was used. The total volume of medium in bioreactor was 10 mL, with a perfusion speed of 0.6 mL/min. Medium was exchanged every 2–3 days according to the manufacturer’s instructions (Cellec Biotech). After 14 and 21 days of cultivation, scaffolds were removed from the bioreactor and analyzed.

#### 2.3.3. Hoechst 33342 Staining

After removal from the bioreactor, constructs were rinsed in PBS, fixed in 4% paraformaldehyde, embedded in paraffin, cross-sectioned (5 µm thick), and deparaffinised using xylene and rehydrated through a series of ethanol washes. To visualize the cell proliferation and distribution through the cross section of the scaffolds, slides were stained with cell permeable nuclear stain Hoechst 33342 (Sigma-Aldrich). Prior to staining, all slides were permeabilized in 0.25% Triton x-100 (Sigma-Aldrich) for 10 min, followed by washing in PBS. Slides were incubated with 1 µg/mL Hoechst for 1 min in the dark and rinsed in PBS, followed by mounting in glycerol/PBS (1:1). Fluorescent staining of cell nuclei was observed under fluorescence microscope (Olympus BX51, Tokyo, Japan).

#### 2.3.4. Histological Analysis

After removal from the bioreactor, constructs were rinsed in PBS, fixed in 4% paraformaldehyde, embedded in paraffin, cross-sectioned (5 µm thick), and stained with H&E (hematoxylin–eosin). All sections were deparaffinised using xylene, and rehydrated through a series of ethanol washes prior to staining. Detection of calcium in tissue sections was done using alizarin red S and von Kossa staining. Slides were stained with alizarin red solution (2%, pH 4.4) for 2 min. For von Kossa staining, slides were incubated with 5% silver nitrate solution in a clear glass coplin jar and placed under UVB light emitting 312 nm (UVItec Ltd., Cambridge, UK) for 2.5 min. Unreacted silver was removed with 5% sodium thiosulfate for 5 min. Mayer’s hematoxylin (Biognost, Zagreb, Croatia) was used as counterstain for 10 min. All slides were dehydrated and mounted with resinous medium (Biognost). Slides were observed under microscope (Olympus BX51).

#### 2.3.5. Isolation of Total Cellular RNA and Real-Time Quantitative Polymerase Chain Reaction (RT-qPCR) Analysis

Total cellular RNA was isolated using TRIzol reagent (Invitrogen, Carlsbad, CA, USA), according to the manufacturer’s instructions. Briefly, tissues were removed from the bioreactor as well as undifferented hMSCs from 2D culture, were washed with cold PBS and homogenized in 1 mL of TRIzol. Tissues from the bioreactor were homogenized with a mixer mill (Retsch, Haan, Germany), while hMSCs were scrapped. After that, 200 µL of chloroform was added followed by centrifugation. Isopropanol was used for RNA precipitation. The RNA concentration was determined by the spectrophotometric method using a NanoVue (Thermo Fisher Scientific) at 260 nm. Total RNA was treated with DNase I (Invitrogen, Carlsbad, CA, USA) to remove genomic DNA and 1 µg of total RNA was reverse transcribed to cDNA using GeneAmp RNA PCR kit (Applied Biosystems, Waltham, MA, USA) according to the manufacturer’s instructions. Reverse transcription was performed in the thermomixer (Eppendorf, Hamburg, Germany) at the following conditions: 10 min at 20 °C, 1 h at 42 °C, 5 min at 99 °C, and 5 min at 5 °C. Relative expression of collagen (Coll), bone sialoprotein (BSP), dentin matrix protein (DMP1), and osteocalcin (OC) were determined by RT-qPCR on the machine 7500 Fast Real-Time PCR System (Applied Biosystems, Foster City, CA, USA) using commercially available primers (Sigma-Aldrich; Table 1) and Power SYBR Green Mastermix (Applied Biosystems). Each reaction consisted of a duplicate in 96-well plates (ABI PRISM Optical 96-Well Plate; Applied Biosystems). The PCR reaction was conducted under the following conditions: 10 min at 95 °C for 1 cycle, 15 s at 95 °C, and 1 min at 60 °C for 40 cycles. The expression levels of osteogenic genes were normalized to β-actin as housekeeping gene, and calculated using the ^ΔΔ^Ct method.

#### 2.3.6. Immunohistochemical Detection of Type I Collagen and Osteocalcin

Sections were deparaffinised, rehydrated, and then subjected to proteinase K (Proteinase K Ready to use, Dako, Togo) antigen retrieval for 12 min at room temperature. Endogenous peroxidase activity was blocked with 3% H_2_O_2_ in PBS for 3–6 min. Nonspecific binding sites were blocked with 10% goat serum (Dako) in PBS for 60 min at room temperature. Sections were then incubated with primary antibody (anti-collagen I, Abcam, Cambridge, UK), diluted 1:400 or anti-osteocalcin (FL-100, Santa Cruz Biotechnology, Dallas, TX, USA) diluted 1:50, respectively, with 1% goat serum in PBS, overnight at 4 °C. After washing, the signal was detected with EnVision Detection Systems Peroxidase/DAB, Rabbit/Mouse (Dako), according to the manufacturer instructions. Hematoxylin was used as a counterstain. Negative controls were processed in the same way with the omittance of primary antibody. Human bone was used as a positive control. Slides were observed under phase-contrast microscope (Olympus BX51).

### 2.4. Statistical Analysis

The experiments were performed in duplicate or more, therefore quantitative results were represented as a mean value with standard deviation. Significant difference between two groups was determined by statistical analysis using one-way ANOVA test, with a *p* < 0.05 value as statistically significant.

## 3. Results

### 3.1. Scaffolds’ Microstructure

**Figure 1:**
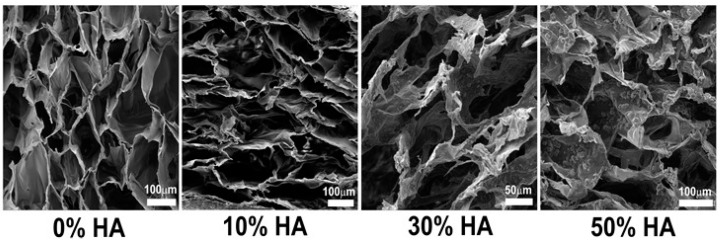
SEM micrographs of cross section of different chitosan-hydroxyapatite (Cht-HA) scaffolds. Pure chitosan scaffold shows smooth surface, while small crystals at the beginning of their growth were found in scaffold with 10% of HA. Petal-like hydroxyapatite crystals are formed in 30% HA scaffold, while cauliflower-like HA particles are homogeneously distributed in scaffold with 50% of HA.

Microscopic analysis of a cross section of different scaffolds indicates a highly porous structure with a visually assessed pore size of up to 200 µm (Figure 1). Comparing the microstructure of pure chitosan scaffold (0% HA), the addition of HA causes alteration of ordered honeycomb-like pore structure. It is not surprising to see such changes in microstructure, taking into account the differences in solution viscosity and crystal size of hydroxyapatite of the different starting solutions. Likewise, the precursors’ amount for corresponding hydroxyapatite fraction affects the final pH value of the composite solution, i.e., the solubility of chitosan that dictates the surface energy between the phases. Shape and orientation of in situ HA crystals are changing by the increase of the HA amount, leading to formation of so-called cauliflower morphology observed at 50% HA scaffold.

### 3.2. Biological Evaluation of Construct (Grafts) Cultured in Perfusion Bioreactor

The regenerative medicine based on stem cell-scaffold culture has already been recognized as a promising alternative for autologous grafts in orthopedic surgery. The capability of differentiation into several cell lineages (osteoblasts, chondrocytes, adipocytes, tenocytes, and myoblasts), immunosuppressive properties, and carrying a lower risk of malignant transformation, makes mesenchymal stem cells more clinically applicable [25]. The presence of calcium phosphate, mainly hydroxyapatite, in chitosan scaffold has shown to increase cell adhesion, proliferation, alkaline phosphatase activity, protein’ adsorption, type I collagen production, and expression of other osteogenic differentiation markers.

To evaluate how different HA fractions affected the cell behavior, such as the cell attachment, we determined the number of attached hMSCs by counting the number of cells in the medium (unattached cells) 24 h after seeding. High percentages of attached cells to scaffolds were confirmed: 93% (±1.80%) for 0% HA, 96.33% (±2.93%) for 10% HA, 91.83% (±1.04%) for 30% HA, and 96.67% (±1.15%) for 50% HA, respectively.

**Figure 2:**
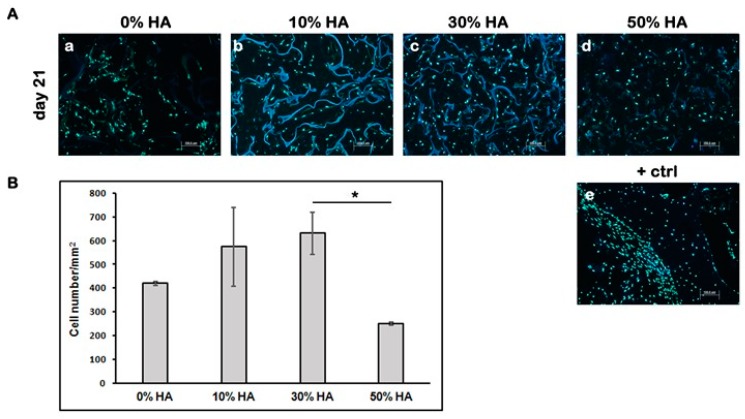
Cell distribution through the cross section of different Cht-HA scaffolds after 21 days of dynamic culture. (**A**) Fluorescence microscopic images of Hoechst stained cells on scaffolds composed of 0% HA (**a**), 10% HA (**b**), 30% HA (**c**) and 50% HA (**d**), respectively. Human bone sample was used as positive control (+ctrl). Representative images are shown at 100× magnification (*n* = 3). Scale bar represents 100 µm. (**e**). (**B**) The cell number/mm^2^ within each Cht-HA scaffold. Significant difference between two groups (*p* < 0.05) is designated as (*).

To visualize the cell distribution through the cross section of the scaffolds, slides were stained with cell permeable nuclear stain Hoechst. The cell number/mm^2^ within each graft was determined after 21 days of dynamic culture by staining of cell nuclei (Figure 2A). The highest cell number/mm^2^ came from grafts seeded on composite scaffolds with 30% HA, while the lowest came from grafts seeded on composite scaffolds with 50% HA (Figure 2B). The results indicate that a high percentage of HA could have unfavorable effects on cell survival during 21 days of dynamic culture.

#### 3.2.1. Histological Analysis of the Grafts

**Figure 3:**
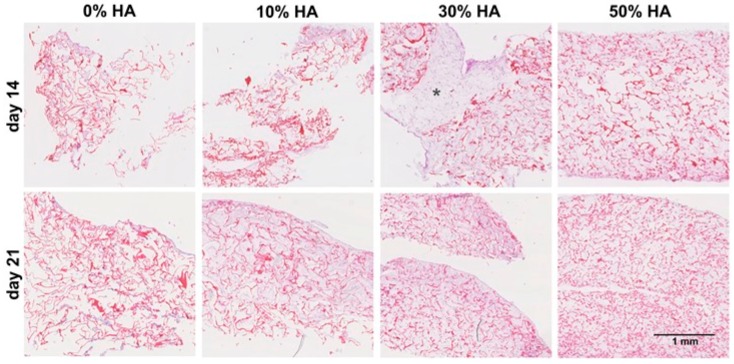
Histology of hMSCs cultured after 14 and 21 days. Hematoxylin-eosin staining of hMSCs-scaffold constructs after 14 and 21 days of culture. Positive influence of 30% of hydroxyapatite can be observed by large amount of newly-formed tissue with respect to the other scaffolds after 14 days of culture, designated by asterisk (*).

Histological analysis of grafts was done after 14 and 21 days of dynamic culture by hematoxylin-eosin staining. Figure 3 points out the significant difference in the amount of newly formed tissue on 30% HA scaffold with uniform cell distribution throughout the entire volume of the graft. Taking a look on a large area of newly formed tissue on aforementioned scaffold (asterisk designation on Figure 3), we can assume that this scaffold’s composition is a suitable environment for hMSCs migration and growth after 14 days of culture.

Continued perfusion in culture has provided further extracellular matrix (ECM) synthesis, especially for constructs seeded on 10% HA scaffold, while the pure chitosan constructs has remained almost the same even after 21 days. It seems that the absence of hydroxyapatite not only influences the osteoinductive properties of chitosan scaffold, yet also the surface properties for cell adhesion and growth. Similar effects were detected on scaffolds with 50% of HA, as indicated by the weaker and non-homogeneous staining. This result is in agreement with lower number of cells counted using Hoechst staining.

#### 3.2.2. Quantitative Evaluation of Osteoinduction

The differentiation program of mesenchymal cells can be divided into three distinct stages regulated by physiological signals and based on different markers expression: proliferation regulated by vitamin D and glucocorticoids; extracellular matrix maturation characterized by alkaline phosphatase activity and extracellular matrix mineralization followed by expression of osteocalcin.

**Figure 4:**
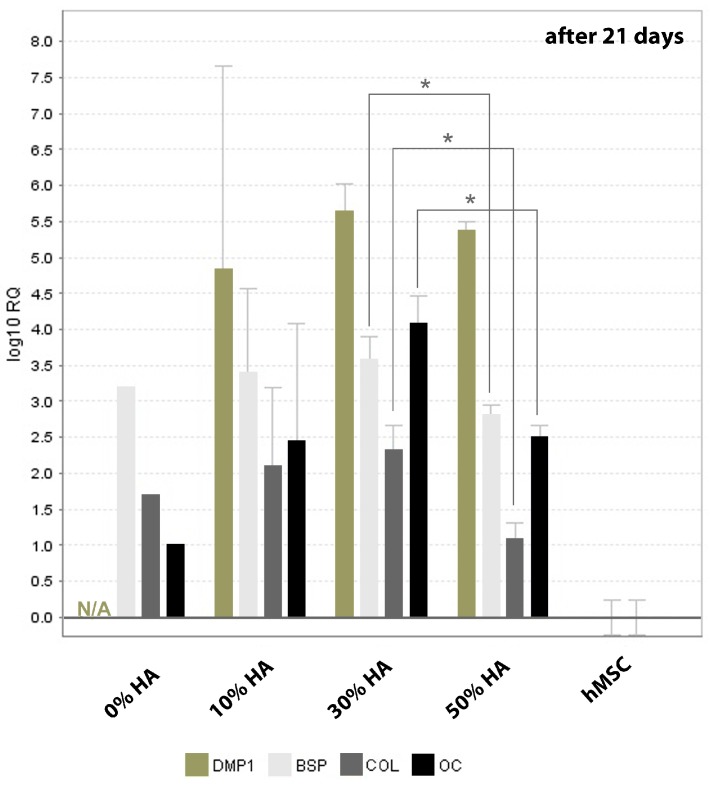
Relative expression (RQ) of osteogenic markers after 21 days of dynamic human mesenchymal stem cells (hMSCs) culture. The relative gene expression was analyzed by comparative cycle threshold method (^ΔΔ^Ct) and the values were normalized to β-actin expression. Significant difference between two groups (*p* < 0.05) is designated as (*).

Figure 4 represents relative expression of specific osteogenic genes on different chitosan-hydroxyapatite scaffolds normalized by the gene expression of hMSCs cultured onto 2D surface, after 21 days of culture. Apart from the dexamethasone-based upregulation of some osteogenic genes, the major impact of hydroxyapatite fraction could be visible at later stage of hMSCs differentiation. Very high deviation of gene expression was found for scaffold with 10% of hydroxyapatite which could be a result of non-homogeneous distribution of smaller in situ formed HA crystals at such low fraction. On contrary, the higher expression of collagen type I, which prepare the extracellular matrix for the onset of mineralization process, was found on 30% HA scaffold regarding pure chitosan and 50% HA scaffold.

Osteocalcin is expressed immediately before the mineralization which categorized it as the late stage marker for osteoblastic differentiation, along with BSP which is highly restricted to bone and mineralized tissues [26]. Accordingly, poor mineralization follows the same trend as collagen I expression, indicated by the lower expression of osteocalcin and BSP detected on scaffolds with lowest and highest HA fraction (0% and 50%). DMP1 represents small integrin-binding ligand linked glycoprotein which is critical for regular bone mineralization [27,28]. With exception of non-detectable expression for 0% HA, all scaffolds did not exhibit significant difference in DMP1 expression after 21 days of culture.

#### 3.2.3. Immunohistochemical Staining of the Grafts

**Figure 5:**
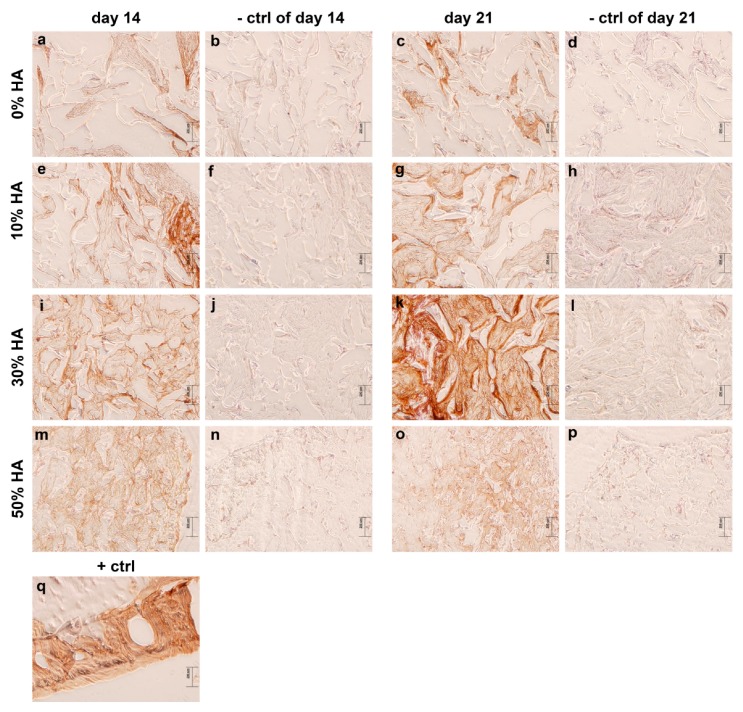
Immunohistochemical examination of collagen I on different scaffolds after 14 and 21 days of osteogenic induction. Brown color indicates positive staining. Weak staining is observed in samples with 0% HA (**a**,**c**). Very strong staining was observed in scaffold composed of 30% HA after 21 days of osteoinduction (**k**). Moderate staining for collagen I was shown for scaffolds composed of 10 and 50% HA (**e**,**g**,**m**,**o**), as well as for scaffold composed of 30% HA after 14 days of osteoinduction (**i**). As negative controls (−ctrl of day 14 and −ctrl of day 21), sections were processed for each sample in the absence of the suitable primary antibody (**b**,**d**,**f**,**h**,**j**,**l**,**n**,**p**). Human bone was used as a sample for positive control (+ctrl) (**q**). Representative images are shown at 100× magnification (*n* = 3). Scale bar represents 200 µm.

The production of type I collagen is one of the earlier events in MSC osteogenic differentiation [29], and it is capable of stimulating MSC osteogenic differentiation through the activation of focal adhesion kinase and extracellular signal-regulated kinase, even without additional ostoinductive factors [30]. Immunohistochemical analysis revealed weak positive staining of collagen I on all constructs after 14 days of culture, indicating early stages of osteoinduction (Figure 5), with the exception of intense localized staining for scaffold with 10% HA. However, remarkably intense and homogeneous staining of type I collagen is observed only on grafts with 30% of hydroxyapatite after 21 days, indicating a greater amount of newly deposited ECM. Enhanced production and expression of collagen and other early stage proteins, including alkaline phosphatase, set the extracellular matrix for initial mineralization. At this point, osteoblasts begin to produce calcium that creates mineralized matrix. Osteocalcin partially plays a role in the regulation of bone mineral deposition, thus its expression can be used as an indication of mineralized matrix formation [31,32,33,34].

**Figure 6:**
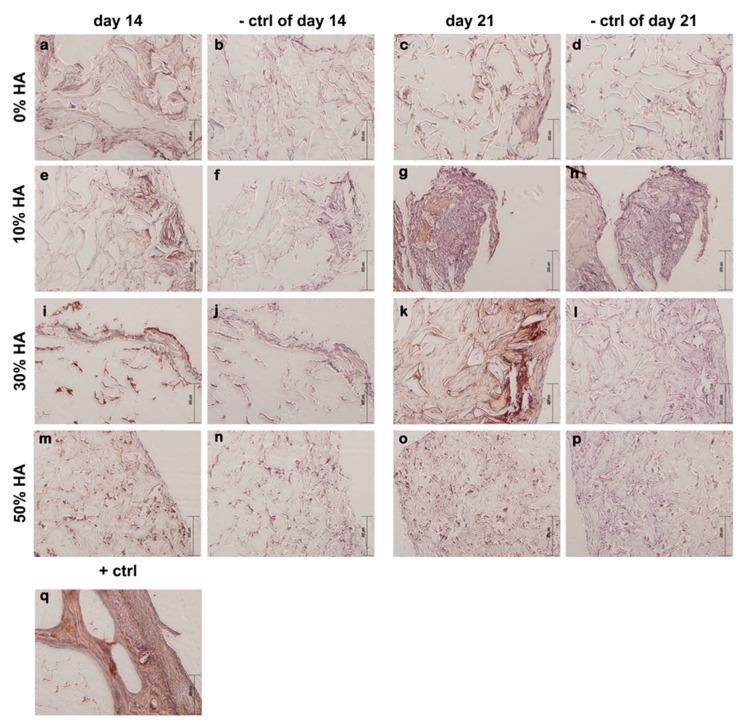
Immunohistochemical staining with anti-ostecalcin. Brown color indicates positive staining. Weak staining is observed in samples with 0% HA (**a**,**c**) and 50% HA (**m**,**o**). Very strong staining was observed in scaffold composed of 30% HA after 21 days of osteoinduction (**k**). Moderate staining for osteocalcin was shown for scaffold with 10% HA (**e**,**g**) as well as for scaffold 30% HA scaffold after 14 days of dynamic culture (**i**). As negative controls (−ctrl of day 14 and −ctrl of day 21), sections were processed for each sample in the absence of the suitable primary antibody (**b**,**d**,**f**,**h**,**j**,**l**,**n**,**p**). Human bone was used as a sample for positive control (+ctrl) (**q**). Representative images are shown at 100× magnification (*n* = 3). Scale bar represents 200 µm.

Immunohistochemistry staining of osteocalcin expression (Figure 6) is in agreement with gene expression determined by RT-qPCR. Scaffolds with 0 and 50% showed insignificant staining after 21 days of culture as compared to the dark brown staining on 30% HA scaffold. Eliminating high interference, weak brown color is detected on 10% HA scaffold, indicating a higher expression of later stage marker with respect to scaffold with 0 and 50% of HA.

Von Kossa and alizarin red S staining were used to characterize the bone nodule formation of hMSCs after 14 and 21 days of dynamic culture. Although Von Kossa staining is nonspecific for calcium ion, it is positive for its carbonate or phosphate precipitates [35], while in the presence of calcium, the staining product of alizarin red S appears red.

**Figure 7:**
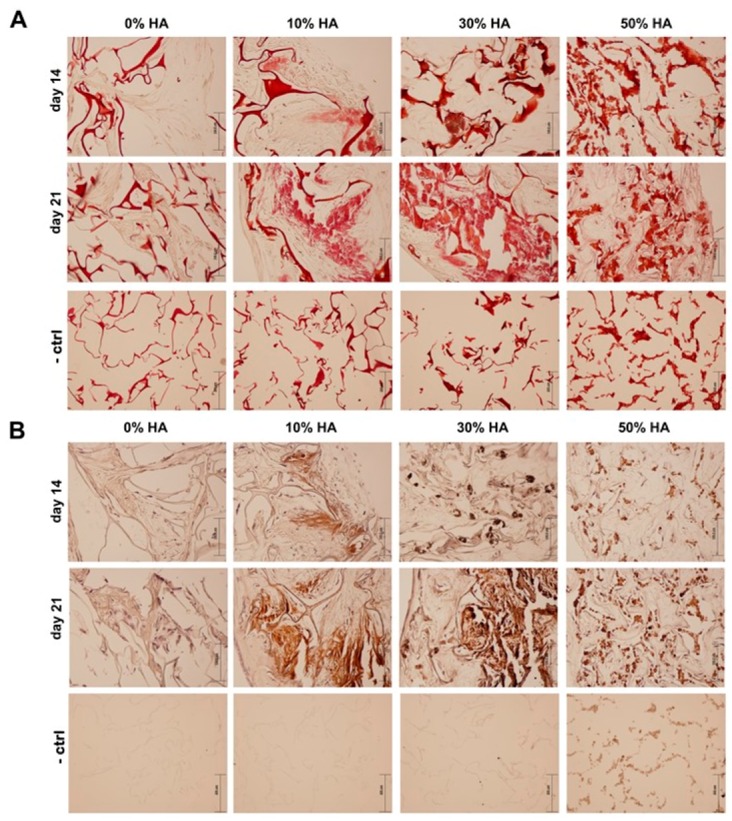
Assessing the mineralization of hMSCs grown in an osteogenic medium within different scaffolds after 14 and 21 days. (**A**) Mineralization of the extracellular matrix with the presence of calcium precipitates was visualized by staining with alizarin red S at days 14 and 21. Positive staining for calcium is signified by red color; (**B**) detection of mineralization using von Kossa staining (including hematoxylin counterstain). Positive staining for calcium phosphate is signified by brown color. Representative images are shown at 200× magnification (*n* = 3) and the scale bars represent 100 µm, respectively. Scaffolds without hMSCs were used as negative controls (−ctrl). Representative images of negative controls are shown at 100× magnification (*n* = 3) and the scale bars represent 200 µm.

Figure 7A,B show induced osteodifferentiation in Cht-HA composite scaffolds, which is significant in the later stage of cell culture. Localized bright staining of mineralized tissue was detected on 10% HA scaffold after 14 days, followed by more intense staining after 21 days of culture. However, mineralized bone nodules stained dark brown were clearly observed in 30% HA scaffold after 21 days, accompanied by strong alizarin red S staining, indicating a greater amount of mineralized matrix. On contrary, 50% HA scaffold exhibited significantly weaker Von Kossa staining, indicating a poor extracellular mineralization as prompted by the highest HA fraction.

## 4. Discussion

Numerous studies focusing on the importance of the preparation technique of potential bone grafts have already been emerged [36]. The in situ synthesis of hydroxyapatite as an osteoinductive inorganic component has been pointed out as a favorable method to reduce nonhomogeneity and potential localized defects in the mechanical performance of the bone graft. The higher homogeneity and better distribution preventing the agglomeration of hydroxyapatite within the sample is crucial in bioresorbability, influencing the local pH gradient of surrounding tissue and finally potential immunological reactions.

Porosity, pore size, and the interconnectivity of scaffolds play a predominant role in cell seeding and proliferation, ingrowth, and vascularization, and biomechanical properties of the scaffold during tissue formation. In fact, those features impact the direction of tissue regeneration. High porosity and pore interconnectivity dictate molecule diffusivity and fluid conductance, especially during tissue healing and integration [37]. Among the first minimum recommended pore size for a scaffold of approximately 100 µm [38], the following studies have shown better osteogenesis on scaffolds characterized with pore sizes larger than 300 µm [39]. It has been shown that relatively larger pores favor direct osteogenesis due to the easier vascularisation and high oxygen permeability, while smaller pores result in osteochondral ossification. However, the type of bone ingrowth depends on the biomaterial features and the geometry of the pores. Even though large pores provide better neotissue penetration, a certain upper limit of porosity and pore size should be considered from a mechanical and biodegradation point of view. Microscopic scanning of prepared Cht-HA scaffolds revealed large irregular pores of up to 200 µm, with good interconnectivity which has been maintained with different HA fractions. Even though pore walls have been disrupted by the increased HA amount, the size of non-defined shape pores and high interconnectivity were maintained. Former studies reported that dynamic cultures performed under stirring and perfusion flow enhance mass transfer, stimulate the cells hydrodynamically or mechanically, and positively influenced cell proliferation [40]. With high porosity, scaffolds can provide unhindered media flow during perfusion conditioning and good cell exposure to the interstitial medium within the whole scaffold, resulting in higher seeding efficiency and an even distribution of cells and newly-formed tissue.

Apart from the existing biomaterials, chitosan as a cationic polymer is extensively utilized in combination with other inorganic ceramics, especially hydroxyapatite, for the further enhancement of tissue regenerative efficacy and osteoconductivity, while chitosan-hydroxyapatite composite materials have exerted favorable bioactivity and bone-bonding ability [41]. Higher bioactivity of chitosan is detected in the presence of hydroxyapatite by the increment of cell number on composite scaffolds. This effect is shown to be limited by the HA fraction, according to the drastic decease of cells on the 50% HA scaffold. One of the important factors involved during cell adhesion and proliferation are the surface properties of the scaffold, including hydrophilicity, surface composition, charge, roughness, topography, etc. Despite bioactivity and biocompatibility, hydroxyapatite is bioresorbable under physiological conditions. More exposed HA crystals observed on 50% HA scaffold under microscopic scanning can impact the rate of HA resorption, i.e., the equilibrium rate between dissolution and precipitation of apatitic crystals during cell culture. This equilibrium could impact local changes in the pH of the medium affecting the cell adhesion and further survival [42]. Although cell seeding was very high for all of the composites, measured by the quantity of detached cells after 24 h of seeding, there is a possibility for cell detachment during culture provoked by the saturation of HA particles. Consequently, lower amounts of remained cells would generate a lower expression of osteogenic genes and poor osteogenesis.

Stem cells are regulated by physical and chemical factors localized in their extracellular surroundings [43]. The MSCs differentiation to bone cells can be induced by exposing the cells to a three-component cocktail of ascorbic acid, β-glycerophosphate, and synthetic steroid, namely dexamethasone. The influence of hydroxyapatite as an osteoinductive component of artificial bone graft has been investigated by numerous studies of hMSCs cell culture in osteogenic and basal environments [44,45,46,47]. Our Cht-HA scaffold with 30% of HA has emerged with an abundant accumulation of fibrous collagen I, and osteocalcin detected by a very strong staining after 21 days of dynamic culture, indicating its advanced osteoinductive potential by activating hMSCs differentiation into osteoblasts.

During osteogenic differentiation, the cells begin to secrete calcium phosphate minerals, referred as the mineralization phase. In this phase, the formation of bone nodule takes place, which is one of the osteodifferentation markers. The extracellular mineralization is considered to be the strongest indicator of osteoblast differentiation and osteogenesis [48]. Intense von Kossa staining in the 30% HA scaffold after 21 days suggests that this microenvironment activates and promotes the production of a mineralized matrix from the cells [49]. Such a high degree of mineralization could indicate an environment conducive to osteogenesis [48].

Biomaterial’s influence on the hMSCs osteodifferentiation is greatly important, especially during in vivo bone restoration. By choosing pertinent biomaterial’s composition, one could achieve simultaneous bone formation and biomaterial’s degradation. We have demonstrated the existence of a critical amount of osteoinductive components, namely hydroxyapatite, for a suitable allogeneic graft development. Poor stem cell differentiation on 50% HA scaffold can be caused by lower number of cells during culture. However, this study shows paramount influence of hydroxyapatite, not only on osteoinductive properties, but also on cell-adhesive surface properties of artificial scaffold as one of primary requirement. Preliminary results presented demonstrate the important impact of hydroxyapatite fraction on hMSCs differentiation that can dictate future development of potential bone substituent, with limitations at a higher HA amount. However, concerning the variability of biological and technical sources, obtained data needs to be compared by additional dynamic cultures of cells derived from different patients.

## 5. Conclusions

The chitosan-hydroxyapatite scaffolds represent a favorable microenvironment for tissue penetration and ingrowth, as indicated by histological analysis of cultured grafts. Extensive influence of hydroxyapatite on hMSC proliferation and osteoinduction can be observed by an emphasized immunostaining of collagen type I and strong osteocalcin and calcium deposit staining on composite scaffolds with 30% of HA, while a higher apatite fraction indicates poor mineralization of hMSCs extracellular matrix. Despite a high percentage of attached cells (90–98%) under perfusion conditioning, poor tissue ingrowth and weak osteogenesis was observed on pure chitosan and 50% HA scaffold, pointing out that chemical composition of scaffolds is one of the dominant factors for good cell seeding and growth. The evaluation of osteonduction markers highlights the impact of hydroxyapatite fractions on osteogenesis, in terms of better ECM mineralization, as promoted by lower amounts (10–30%) of the osteoinductive phase within chitosan-based scaffolds.

## Figures and Tables

**Table 1:** Human primer sequences used for determination of gene expression levels by reverse transcription-polymerase chain reaction analysis.

Primers	5′-	Sequences	Annealing Temperature (°C)
***COL1A1***	forward	GCTATGATGAGAAATCAACCG	61.1
	reverse	TCATCTCCATTTCCAGG	61.6
***IBSP***	forward	GGAGACTTCAAATGAAGGAG	57.9
	reverse	CAGAAAGTGTGGTATTCTCAG	56.4
***DMP1***	forward	CAACTATGAAGATCAGCATCC	58.8
	reverse	CTTCCATTCTTCAGAATCCTC	59.3
***BGLAP***	forward	TTCTTTCCTCTTCCCCTTG	60.8
	reverse	CCTCTTCTGGAGTTTATTTGG	59.3
